# Predictors of Therapy Outcome in Eating Disorders: from Psychopathology to Personality

**DOI:** 10.1192/j.eurpsy.2022.96

**Published:** 2022-09-01

**Authors:** F. Fernandez-Aranda, S. Jimenez-Murcia, R. Granero

**Affiliations:** 1 University Hospital of Bellvitge,-IDIBELL, Department Of Psychiatry, Barcelona, Spain; 2 Hospital Universitario de Bellvitge, Psiquiatría, Bracelona, Spain; 3 School of Medicine and Health Sciences, University of Barcelona, Department Of Clinical Sciences, Barcelona-Campus Bellvitge, Spain; 4 University Hospital of Bellvitge-IDIBELL and CIBERobn, Department Of Psychiatry, Barcelona, Spain; 5 University Autonoma Barcelona, Department Of Psychobiology And Methodology, Bellaterra, Spain

**Keywords:** Eating Disorders, Psychotherapy, predictors, personality

## Abstract

Eating disorders are severe mental disorders, with high mortality rates and high incidence in adolescence and early adulthood, especially in women. The course of these disorders is uncertain and treatment outcomes are limited. Several factors such as duration of the disorder, dysfunctional personality traits and cognitive profiles, as well as genetic vulnerabilities, will influence adherence and response to treatment. In this presentation we will include recent results on prospective observational studies, analyzing personality and cognitive predictors of treatment response in eating disorders, as well as potential associated neurobiomarkers.

**Disclosure:**

FFA received consultancy honoraria from Novo Nordisk and editorial honoraria as EIC from Wiley. The rest of the authors declare no conflict of interest. The funders had no role in the design of the study; in the collection, analyses, or interpretation of

